# Dimensional Variations of Left-Sided Double-Lumen Endobronchial Tubes

**DOI:** 10.1155/2019/3634202

**Published:** 2019-09-24

**Authors:** Niels Hegland, Sebastian Schnitzler, Jan Ellensohn, Marc P. Steurer, Markus Weiss, Alexander Dullenkopf

**Affiliations:** ^1^Institute for Anesthesia and Intensive Care Medicine, Spital Thurgau AG, Frauenfeld, Switzerland; ^2^Department of Anesthesia and Perioperative Care, University of California, San Francisco, USA; ^3^Department of Anesthesia and Children's Research Centre, University Children's Hospital, Zurich, Switzerland

## Abstract

**Background:**

Tube size selection is critical in ventilating patients' lungs using double-lumen endobronchial tubes (DLTs). Little information about relevant parameters is readily available from manufacturers. The aim of this study is to provide reference data for relevant dimensions of conventionally available DLTs.

**Methods:**

In this study in a benchmark in vitro setup, several dimensional parameters of four sizes of left-sided double-lumen endobronchial tubes from six different manufacturers were assessed, such as distances and diameters of tube shaft, cuff lengths, and diameters as well the angle at the tip.

**Results:**

Endobronchial tubes of ostensibly the same size revealed wide variation in measured parameters between brands from different manufacturers. In some parameters, there was an overlap between different sizes from the same manufacturer, i.e., diameters and distances did not increase with increasing nominal endobronchial tube size. The information about dimensions of endobronchial tubes provided by manufacturers' leaflets is insufficient.

**Conclusions:**

Endobronchial tube size selection carries unnecessary uncertainty because clinically relevant parameters are unknown and vary considerably between different manufacturers.

## 1. Introduction

One-lung ventilation for thoracic surgery and occasionally also in critical care medicine represents one of the most challenging airway and ventilation management tasks for anesthesiologists and/or intensivists [1, 2]. The bronchial blockers and double-lumen endobronchial tubes (DLTs) are the two main techniques for isolation of the lungs and one-lung ventilation, with the DLT used more frequently [3–5]. Irrespective of the decision to use a left-sided or right-sided DLT, choosing the appropriate size is critical. The consequences of inserting/using an inappropriately sized DLT can result in significant clinical problems, such as difficulty in ventilating the lungs' potential difficulties in letting the lung collapse, the need for tube exchange with reintubation, severe injuries to the airway, impaired surgical conditions, or even inability to perform the planned surgical procedure [2, 6–9].

Strategies for the actual sizing vary from the “one size fits almost all” approach to selection of a DLT that most closely matches the patient's airway anatomy with deflated cuffs [2]. Currently, there are two main approaches to the selection of the correctly sized DLT for a given patient when considering patient anatomical conditions. First, reference tables are used that indicate the DLT size based on a patient's height and gender [2, 9, 10]. Second, there are methods incorporating actual patient information from radiological imaging as tracheal width or the size of the left main-stem bronchus [2, 11–16]. The latter has become more relevant in recent years, as the majority of patients planned for thoracic surgery with one-lung ventilation have usually undergone preoperative computed tomography (CT) imaging of their lungs and trachea-bronchial tree.

However, the size selection of the outer diameter and length of a DLT remains challenging even with the availability of precise CT-based knowledge of individual anatomical dimensions. This is further complicated by the fact that the effective outer diameter of a DLT is indicated as circumference on the packaging as “French” (*1 French (Fr)* = *1 Charriere (Ch)* = *1/3 mm*) and moreover varies throughout the length of the tube. For the proper choice of a DLT, further dimensions such as the outer diameter of its endobronchial portion and various section lengths are important. However, they are not indicated in the manufacturers' leaflets [11, 17, 18].

It is the aim of this work to present a point of reference for the relevant dimensions of conventionally available DLTs.

## 2. Materials and Methods

Left-sided DLTs of the sizes 35 Fr, 37 Fr, 39 Fr, and 41 Fr from six different manufacturers were included in this in vitro study (Table 1).

**Figure 1:**
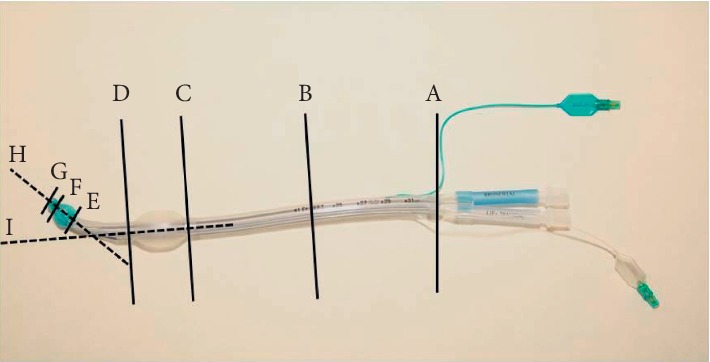
Schematic of localization of measured parameters. A = beginning of the DLT shaft. B = midpoint between the beginning of the shaft of the tube (A) and the proximal edge of the tracheal cuff (C). C = proximal edge of the tracheal cuff. D = distal edge of the tracheal cuff. E = proximal edge of the bronchial cuff. F = distal edge of the bronchial cuff. H = virtual extrapolated longitudinal line through the middle of the tracheal tube shaft. I = virtual extrapolated longitudinal line through bronchial tube shaft.

One brand new DLT of each type and size was measured twice by two investigators (NH and SS), i.e., four measurements of each dimensional parameter were taken. The investigators were performing their measurements independently and not knowing about the results obtained by their counterpart. Dimensions of the tube (diameter and length), cuff (diameter and length), and the angle of the endobronchial tube portion were determined (Figure 1). All measurements were performed with the intubation stylet removed from the DLT. For all measurements, a commercially available ruler (measurement accuracy 1 mm), a sliding caliper (measurement accuracy of 0.1 mm), an orthopedic protractor, 10 ml syringes (B. Braun, Melsungen, Germany) for inflating the cuffs, and a manual cuff pressure manometer (Rüsch Endotest®; Teleflex, Athlone, Ireland) to adjust cuff pressure were used.

The following dimensional parameters of the DLT were measured or calculated (Figure 1):

 Outer tube diameter (OD, always both: lateral and anterior-posterior) is as follows:
   OD_MIDDLE_ = diameter in the center (B) between the beginning of the shaft of the tube (A) and the proximal edge of the tracheal cuff (C)
   OD_PTC_ = diameter directly at the proximal edge of the tracheal cuff (C)
   OD_DTC_ = diameter directly at the distal edge of the tracheal cuff (D)
   OD_PBC_ = diameter directly at the proximal edge of the bronchial cuff (E)
   OD_DBC_ = diameter directly at the distal edge of the bronchial cuff (F)
 Internal tube diameter (ID): The tracheal and endobronchial lumen of all investigated DLTs were assessed for passability of a fiber-optic bronchoscope (Karl Storz, Germany) with diameters 5.5 mm and 4.0 mm, respectively.
 Lengths are as follows:
   Length A–C = distance from beginning of DLT shaft (A) to the proximal edge of the tracheal cuff (C)
   Length C-D = length of the tracheal cuff
   Length D-E = distance from the distal edge of the tracheal cuff (D) to the proximal edge of the bronchial cuffs (E)
   Length E-F = length of the bronchial cuff
   Length F-G = distance from the distal edge of the bronchial cuffs (F) to the tip of the tube (G)
   Length A–G = tube length from bifurcation (A) to the tip of the tube (G)
 Outer cuff diameters: outer cuff diameters were assessed with the cuffs not being in contact with a model of tracheal/bronchial lining. For the measurement of the tracheal and bronchial cuff diameters, the respective cuff inflation line was connected via a three-way stopcock to the manual cuff pressure gauge and inflated with air to the target pressure of 20 H_2_O using a 10 ml syringe. Upon reaching the target pressure, the three-way stopcock was closed so that no air could escape. The volume of air necessary to generate the target pressure of 20 H_2_O was noted.
 Angle is as follows:
   Angle H-I = mediastinal angle between a virtual longitudinal line through the middle of the tube shaft and the bronchial tube section

Since DLTs rarely present with a truly round cross-sectional area but are rather elliptically shaped, the cross-sectional area (CSA) was calculated at the points indicated below, utilizing the measured width and height of the tube according to the formula $\text{CSA}=\pi \;*\;\text{width}/2\left(=\text{lateral}\right)*\text{height}/2\left(=\text{anterior}‐\text{posterior}\right)$.

 CSA_MIDDLE_ = cross-sectional area of the endobronchial tube in the middle (B) between the beginning of the tube shaft (A) and the proximal edge of the tracheal cuff (C)
 CSA_PTC_ = cross-sectional area of the endobronchial tube directly at the proximal edge of the tracheal cuff (C)
 CSA_DTC_ = cross-sectional area of the endobronchial tube directly at the distal edge of the tracheal cuff (D)
 CSA_PBC_ = cross-sectional area of the endobronchial tube directly at the proximal edge of the bronchial cuff (E)
 CSA_DBC_ = cross-sectional area of the endobronchial tube directly at the distal edge of the bronchial cuff (F)

For all DLTs investigated, it was noted whether the package insert provides information on the abovementioned parameters besides the nominal tube size given in French (1/3 mm).

## 3. Calculations and Statistics

Descriptive statistics were used to compare measured data. All data are presented as median (minimum-maximum). The analysis was performed using Microsoft Excel 2010 (Microsoft, Redmond, USA).

## 4. Results

A total of 624 measured or calculated values were obtained from the 24 different DLTs.

Outer diameters measured and cross-sectional areas calculated are expressed in Tables 2 and 3. As can be seen in Table 3, certainly the bronchial part is narrower than the tracheal part. Regarding the latter, the narrowest part (looking at the three assessed sections) was the middle of the tube shaft in all investigated DLTs and sizes. One DLT (VIVASIGHT-DL®) in all sizes demonstrated the largest cross-sectional area proximal to the tracheal cuff, while another one (Portex Blue Line® Endobronchial Tube) was so distal to the tracheal cuff. In all other DLTs, the largest CSA varied with DLT size. The largest variations in CSA were found close to the tube tip; that is, for the CSA_DBC_ in 37 Fr-sized endobronchial tubes, the smallest CSA was 83% of the largest.

For the majority of the DLT types and sizes examined, the respective CSA increased appropriately with increasing size of the DLT. But remarkably, in 4 constellations, the CSA of the next smaller DLT from the same manufacturer was larger than in its next larger size.

All endobronchial lumen were easily passable by using a fiberoptic bronchoscope with an ID of 4.0 mm, as were all tracheal lumen. Neither lumen of the DLTs sizes 35 and 37 was passable by using a fiberoptic bronchoscope with an ID of 5.5 mm. Passability with an ID 5.5 mm bronchoscope was given only for the tracheal lumen of the VIVASIGHT-DL® and both lumina of the Hudson RCI® Sheridan® SHER-I-BRONCH® Endobronchial Tube in size 39 Fr. In size 41 Fr DLTs, both lumina of the VIVASIGHT-DL® and the Well Lead® endobronchial tube were easily passable, as was the bronchial lumen of the Mallinckrodt® endobronchial tube. In the Hudson RCI® Sheridan® SHER-I-BRONCH® endobronchial tube and the Portex Blue Line® endobronchial tube, both lumina were tight fitting but passable, and neither lumen of the Rüsch Bronchopart® was passable with the ID 5.5 mm bronchoscope.

The length measurements varied more among the various manufacturers and DLT sizes than did the CSAs (Table 4). Surprisingly, in no DLT model were all the distances of the next larger DLT size longer than the ones of the corresponding next smaller DLT model. The shortest distance from the DLT bifurcation to the proximal edge of the tracheal cuff (A–C) was 66% of the longest distance (35 Fr). The shortest tracheal cuff was 73% of the longest (35 Fr). The bronchial cuff length differed by as much as 51% (shortest to longest; 41 Fr). The most inconsistent measurement was the distance from the distal end of the bronchial cuff to the tube tip (F-G; 28% shortest to longest; 35 Fr). Overall (A–G), the shortest DLT was only 72% of the longest (35 Fr).

Tracheal cuff dimensions revealed the largest variations in OD for a given DLT size of 72% (smallest to largest) for tracheal cuffs and 54% for bronchial cuffs (41 Fr) (Table 5). The volume of air necessary to generate a cuff pressure of 20 cm H_2_O varied from 1.3 ml (0.8–1.5; Rüsch Bronchopart®, size 37 Fr) to 9.5 ml (9.0–10.3; Portex Blue Line® Endobronchial Tube, size 41 Fr) in bronchial cuffs and from 6.3 ml (5.8–6.5; Hudson RCI® Sheridan® SHER-I-BRONCH® Endobronchial Tube, size 35 Fr) to 22.6 ml (22.0–24.0; Portex Blue Line® Endobronchial Tube, size 41 Fr).

The most impressive and surprising variation was that of the measured angle between the DLT shaft and the bronchial portion (Table 6), with the largest angle being more than three times that of the smallest for a given DLT size (35 Fr).

The package insert for the Portex Blue Line® DLT was the only one that included additional information regarding the DLT's dimensions (tracheal and bronchial cuff diameter in the uninflated state).

## 5. Discussion

This study investigated the dimensional design of left-sided double-lumen endobronchial tubes in frequently used DLT sizes produced by six different manufacturers, including the recently introduced VIVASIGHT® DLT.

The most important finding was that there is considerable variability in the majority of all measured parameters among similarly sized DLT brands. The reported parameters may assist in further studies about the question which size of a double-lumen endobronchial tube to use in a certain patient.

In this study, five commonly used conventional left-sided DLTs as well as the new VIVASIGHT® DLT were examined. The VIVASIGHT® includes an integrated video camera and illuminating system that provides assistance with tube placement and continuous visual surveillance during the procedure [1]. The integrated camera is placed just below the tracheal cuff. As a result, the VIVASIGHT® has a larger cross-sectional area (CSA) down to that point. Apart from that finding and from the Portex Blue Line® DLTs demonstrating the largest cross-sectional areas proximal to the tracheal cuff, it is not possible to give a synthesis in what brands of DLTs are consistently larger or smaller than the majority, because of the intrabrand variation.

The parts of the airway most relevant for the tube size selection are the larynx and the main bronchi, with the trachea presenting less of a problem with regard to tube diameters. [2, 11, 14, 15, 17–19] The more distal parts of the patients' airways quickly become anatomically narrower and shorter, making the corresponding (more distal; patient end) portions of the respective DLT critical for proper airway management [1]. As such, the distance from tracheal to bronchial cuff, the length of the bronchial cuff, and the tube diameter in the bronchial segment can be regarded as particularly important [18]. The length of DLT parameters in relation to the patient's height is important for tube size selection, as is the angle of the bronchial lumen for insertion of a left-sided DLT. Our results confirm the measurements of Watterson and Harrison [17] and Russell Strong [18]. Over 20 years ago, these researchers observed considerable differences between similarly sized DLTs delivered by different manufacturers with regard to the length of the endobronchial segments of DLTs. In addition to the measurements conducted by Russell and Strong [18], we used calculated CSAs in accordance with the formula for elliptical shapes rather than the circular formula because both the tracheal and bronchial segments of a DLT do not represent circles. We further included additional parameters and tracheal tube brands, as Russell and Strong [18] mainly focused on the bronchial cuff and did their study in 2003. Comparable to our results, the Portex DLT was the one with the longest bronchial cuff.

In contrast to conventional tracheal tubes, whose nominal size is defined by the internal diameter (with possibly varying outer diameters), the nominal size of endobronchial tubes, according to the International Organization for Standardization (ISO 16628), is defined by the outer diameter of the (tracheal) tube shaft. However, for anesthetists, it is not common knowledge where exactly on the tube shaft the diameter is taken.

In clinical practice, anesthetists or intensivists have learnt to live with these limitations for years, even though the problems associated with the use of (inappropriate) DLTs are considerable. Clayton-Smith et al. [8] reported an incidence of airway injuries of close to 30%, and Heir et al. [20], in a study, including the VIVASIGHT® DLT reported a rate of DLT dislodgement during positioning in almost 2/3 and during surgery in almost 1/3 of their studied patients, respectively. Nevertheless, there is little to no access to the critical DLT dimensions (i.e., by package insert), despite the call for such detail to be more readily available, which was issued over 20 years ago [18]. Most clinicians probably assume that the various DLT products are highly comparable and standardized in size with relevant dimensions becoming proportionally larger with increasing DLT formal size. However, this is not always the case, as shown in our study by the overlap of certain dimensions among different DLT sizes from the same manufacturer. This was similarly demonstrated by Russell and Strong before and has unfortunately not changed since then [18]. So, despite the fact that more information about a given patient's anatomy is readily available (CT scans) nowadays, correctly matching it is still almost impossible because of the lack of knowledge about a given DLT's dimensions. Without knowledge of tube proportions, approaches based solely on the anatomy and dimensions of the tracheal and/or the left main bronchus, such as those described by Brodsky et al. [11, 15], will still involve a great deal of inaccuracy and uncertainty. Reference tables provided by manufacturers, e.g., on dedicated websites, could be of value here, e.g., in patients with unusually long or short left main bronchus, as assessed by CT scan.

An important point to be mentioned is the large variability in the volume of air necessary to generate a cuff pressure of 20 cm H_2_O in both the bronchial and the tracheal cuff. The use of a cuff pressure manometer in clinical practice is therefore recommended.

A limitation of our investigation is the fact that we only measured one copy of each DLT size and manufacturer/model. Russell and Strong showed in a similar study in 2003 that even nominally identical DLTs of the same manufacturer and size can reveal considerable variation in their dimensions, especially in the bronchial segment [18]. The calculation of the CSA according to the stated formula is an approximation. Given the noncircular cross-sectional shapes of the DLTs, the inner diameter could have been estimated digitally from high-resolution CT scans. We further did not assess the clinical consequences of the size variations (i.e., resistance) in a physical model with anatomic variations or in a clinical setting. Most importantly, it is not clear what degree of variation really presents a clinical problem, because, other than in children [21], there are no data on the anatomic parameters in adults that would predispose them for given DLT sizes. Being beyond the scope of this work, the ultimate clinical goal maybe would be to obtain data about the rate of necessary tube changes when following different guidelines for tube size selection.

## 6. Conclusions

Considerable dimensional differences among similarly sized DLTs from different manufacturers are present. Inconsistent proportionalities with increasing DLT size and incomplete declaration of relevant dimensions for individual DLTs can limit the proper selection of a suitable DLT for one-lung ventilation and should be made more easily available. The new DLT model VIVASIGHT® with an integrated camera has a considerably larger cross-sectional area compared to similarly sized DLT brands.

## Figures and Tables

**Table 1:** Investigated left-sided double-lumen endobronchial tubes (sizes 35 F, 37 F, 39 F, and 41 F).

Brand	Manufacturer
Hudson RCI® Sheridan® SHER-I-BRONCH® endobronchial tube	Teleflex, Athlone, Ireland
Mallinckrodt® endobronchial tube	Covidien, Tullamore, Ireland
Portex Blue Line® endobronchial tube	Smiths Medical, Hythe, UK
Rüsch Bronchopart®	Teleflex, Athlone, Ireland
VIVASIGHT-DL®	ETView Medical, Misgav, Israel
Well Lead® endobronchial tube	Well Lead Medical, Panyu, China

**Table 2:** Outer diameter (OD), presented as lateral diameter (lat) und antero-posterior diameter (ap) of investigated left-sided double-lumen endobronchial tubes (DLT).

Parameter	Size (Fr)	Axis	Sheridan DLT	Mallinckrodt DLT	Portex DLT	Rüsch DLT	VIVASIGHT DLT	Well Lead DLT
OD_MIDDLE_	35	lat	13.0 (13.0–13.3)	12.7 (12.7–12.7)	12.4 (12.4–12.7; ^*∗*^94%)	12.5 (12.4–12.6)	13.2 (12.9–13.5)	12.9 (12.4–13.5)
		ap	11.3 (11.0–11.5)	11.7 (11.7–11.8)	10.9 (10.8–11.0; ^*∗*^93%)	11.0 (10.9–11.2)	11.7 (11.6–11.9)	11.6 (11.5–12.0)
	37	lat	13.0 (12.5–13.6; ^*∗*^94%)	13.3 (13.3–13.5)	13.3 (13.0–13.5)	13.5 (13.0–13.8)	13.4 (12.9–13.9)	13.8 (13.6–14.0)
		ap	11.2 (10.1–11.5; ^*∗*^85%)	12.7 (12.6–12.7)	11.4 (11.2–11.5)	11.4 (11.3–11.6)	13.2 (12.7–13.8)	13.1 (13.0–13.4)
	39	lat	14.4 (14.3–14.6)	14.4 (14.4–14.5)	13.7 (13.5–14.0)	13.4 (12.8–14.0; ^*∗*^93%)	14.3 (14.1–14.3)	14.3 (14.0–14.7)
		ap	12.0 (11.9–12.2)	13.5 (13.0–14.0)	11.8 (11.8–11.9; ^*∗*^87%)	12.5 (12.4–14.5)	13.1 (13.1–13.2)	13.6 (13.3–13.8)
	41	lat	14.5 (14.2–14.7)	14.8 (14.7–14.9)	14.5 (14.3–14.7; ^*∗*^96%)	14.4 (14.3–14.7)	15.0 (15.0–15.0)	14.9 (14.8–15.0)
		ap	12.8 (12.7–12.8)	13.8 (13.6–13.9)	12.5 (12.3–12.6; ^*∗*^89%)	13.0 (13.0–13.1)	14.0 (14.0–14.0)	14.0 (13.9–14.2)

OD_PTC_	35	lat	13.7 (13.5–13.9)	12.8 (12.7–12.8)	12.8 (12.2–13.3)	12.5 (12.3–12.6; ^*∗*^91%)	13.5 (13.2–13.7)	13.3 (13.0–13.5)
		ap	11.8 (11.7–11.9)	12.1 (12.1–12.1)	11.6 (11.6–11.9; ^*∗*^93%)	11.7 (11.6–11.7)	12.5 (12.3–12.7)	12.4 (12.1–13.0)
	37	lat	13.9 (13.5–14.3)	13.4 (13.3–13.6; ^*∗*^91%)	14.0 (13.6–14.0)	13.8 (13.5–13.9)	14.7 (14.6–15.0)	14.2 (13.8–14.6)
		ap	12.0 (11.9–12.2; ^*∗*^87%)	13.2 (13.1–13.2)	12.0 (12.0–12.1; ^*∗*^87%)	12.0 (11.9–12.0; ^*∗*^87%)	13.7 (13.5–13.9)	13.8 (13.4–14.3)
	39	lat	14.7 (14.4–15.0)	14.2 (14.1–14.3)	14.2 (13.9–14.7)	13.9 (13.1–14.5; ^*∗*^94%)	14.8 (14.7–14.9)	14.8 (14.5–15.0)
		ap	12.7 (12.7–12.8; ^*∗*^89%)	13.4 (13.4–13.5)	12.7 (12.5–12.8; ^*∗*^89%)	13.0 (12.9–13.1)	14.3 (13.8–14.9)	14.1 (13.8–14.7)
	41	lat	14.8 (14.5–15.0)	14.9 (14.8–14.9)	15.2 (15.0–15.5)	14.7 (14.4–14.7; ^*∗*^94%)	15.6 (15.5–16.1)	15.6 (15.1–15.9)
		ap	13.5 (13.4–13.6)	14.0 (14.0–14.1)	13.3 (13.3–13.4; ^*∗*^91)	13.7 (13.6–13.7)	14.6 (14.3–14.9)	14.6 (14.6–14.7)

OD_DTC_	35	lat	14.6 (14.2–14.9)	12.9 (12.8–13.0; ^*∗*^88%)	13.1 (12.8–13.2)	13.1 (13.1–13.2)	13.9 (13.8–14.1)	13.4 (12.5–13.5)
		ap	11.8 (11.8–12.0)	12.1 (12.1–12.2)	11.6 (11.6–12.1; ^*∗*^89%)	11.6 (11.6–11.7; ^*∗*^89%)	13.1 (12.8–13.5)	12.5 (12.1–13.3)
	37	lat	13.6 (13.6–14.2; ^*∗*^93%)	13.6 (13.6–13.8; ^*∗*^93%)	13.6 (13.6–14.0; ^*∗*^93%)	13.8 (13.4–14.0)	14.6 (14.4–14.7)	14.3 (14.1–14.5)
		ap	12.2 (12.1–12.3)	13.2 (13.2–13.2)	12.0 (11.9–12.2)	11.9 (11.7–12.2; ^*∗*^84%)	14.2 (13.8–14.7)	13.9 (13.5–14.2)
	39	lat	14.8 (14.7–14.9)	14.5 (14.4–14.6)	14.3 (14.0–14.4; ^*∗*^97%)	14.4 (14.2–14.6)	14.8 (14.7–15.0)	14.7 (14.4–15.0)
		ap	12.8 (12.8–12.9)	13.4 (13.3–13.5)	12.6 (12.6–12.7; ^*∗*^90%)	12.9 (12.7–13.4)	14.0 (13.9–14.4)	14.0 (13.8–14.3)
	41	lat	15.3 (15.0–15.4)	14.9 (14.8–15.0)	15.1 (15.0–15.2)	14.7 (14.6–14.7; ^*∗*^94%)	15.4 (15.1–15.5)	15.6 (15.3–15.6)
		ap	13.6 (13.5–13.7)	14.0 (14.0–14.1)	13.2 (13.1–13.6; ^*∗*^90%)	13.5 (13.5–14.1)	14.6 (14.5–14.7)	14.5 (14.4–14.6)

OD_PBC_	35	lat	8.8 (8.3–9.0)	9.0 (8.9–9.1)	7.8 (7.5–8.1; ^*∗*^85%)	9.2 (9.1–9.3)	9.0 (8.5–9.5)	9.0 (8.7–9.1)
		ap	10.8 (10.7–10.8)	9.5 (9.4–9.7; ^*∗*^88%)	10.7 (10.6–10.9)	10.7 (10.5–10.9)	10.4 (10.3–10.5)	10.3 (10.2–10.6)
	37	lat	8.8 (8.5–9.1)	10.0 (9.5–10.0)	7.8 (7.7–8.0; ^*∗*^78%)	9.3 (9.1–9.7)	8.9 (8.8–9.0)	9.8 (9.6–9.9)
		ap	11.1 (10.8–11.2)	10.8 (10.7–11.0)	10.9 (10.7–11.0)	10.8 (10.7–11.2)	10.7 (10.5–11.3; ^*∗*^96%)	11.1 (11.0–11.2)
	39	lat	8.7 (8.5–9.0)	10.4 (10.3–10.8)	8.1 (7.8–8.4; ^*∗*^78%)	10.4 (9.8–10.8)	9.3 (8.9–9.7)	9.7 (9.3–10.3)
		ap	11.3 (11.1–11.5)	10.9 (10.8–11.1)	11.7 (11.2–11.8)	11.6 (11.4–11.7)	10.8 (10.5–11.0; ^*∗*^92%)	11.4 (11.0–12.0)
	41	lat	8.9 (8.8–9.4)	10.2 (10.1–10.4)	8.8 (8.7–9.5; ^*∗*^82%)	10.7 (10.0–11.1)	9.1 (8.9–9.2)	9.9 (9.2–10.0)
		ap	12.5 (12.2–12.7)	11.2 (11.0–11.5)	12.3 (12.2–12.4; ^*∗*^90%)	12.1 (11.9–12.2)	12.2 (12.1–12.3)	11.7 (11.5–11.9)

OD_DBC_	35	lat	9.0 (8.5–9.3)	9.1 (9.1–9.2)	8.4 (8.0–8.6; ^*∗*^89%)	9.3 (9.1–9.7)	9.4 (9.0–9.8)	8.7 (8.4–9.1)
		ap	11.2 (10.9–11.3)	10.2 (10.1–10.3)	9.9 (9.6–10.0; ^*∗*^88%)	10.1 (9.5–11.0)	11.0 (10.8–11.0)	10.7 (10.3–10.9)
	37	lat	9.8 (9.7–10.0)	10.0 (10.0–10.0)	8.6 (8.5–8.7; ^*∗*^86%)	9.9 (9.3–10.3)	9.2 (9.1–9.6)	9.9 (9.6–10.0)
		ap	11.4 (11.4–11.9)	11.1 (11.0–11.3)	10.0 (10.0–10.1; ^*∗*^84%)	11.4 (10.6–11.5)	11.4 (11.3–11.5)	11.9 (11.6–12.0)
	39	lat	8.9 (8.7–9.0; ^*∗*^83%)	10.5 (10.0–10.5)	9.6 (9.2–9.7)	10.2 (10.1–10.5)	10.2 (10.0–10.3)	10.7 (10.0–12.0)
		ap	11.7 (11.7–11.8)	11.5 (11.0–11.6)	11.4 (11.3–11.5)	11.5 (10.9–11.8)	11.3 (11.3–11.3)	10.7 (9.7–11.8; ^*∗*^91%)
	41	lat	8.9 (8.7–9.2; ^*∗*^84%)	10.6 (10.5–10.7)	10.0 (9.9–10.0)	10.6 (10.3–10.7)	9.6 (9.2–9.7)	10.2 (9.8–10.5)
		ap	12.8 (12.5–12.9)	11.5 (11.4–11.7)	11.3 (11.0–11.5; ^*∗*^88%)	12.3 (12.0–12.6)	12.0 (12.0–12.1)	12.0 (12.0–12.3)

OD_MIDDLE_ = outer diameter in the middle (B) between the beginning of the DLT shaft (A) and the proximal edge of the tracheal cuff (C); OD_PTC_ = outer diameter at the proximal edge of the tracheal cuff (C); OD_DTC_ = outer diameter at the distal edge of the tracheal cuff (D); OD_PBC_ = outer diameter at the proximal edge of the bronchial cuff (E); OD_DBC_ = outer diameter at the distal edge of the bronchial cuff (F) (see also Figure 1). DLT sizes are in French (= 1/3 mm). Data are displayed as median (minimum-maximum), and dimension is millimeter (mm). The largest diameter of a respective DLT size is shaded in gray (100%), the smallest diameter marked with an asterisk (^*∗*^) (proportion of largest OD).

**Table 3:** Calculated cross-sectional areas (CSAs) of investigated left-sided double-lumen endobronchial tubes (DLTs) based on measurements expressed in Table 2.

Parameter	Size (Fr)	Sheridan DLT	Mallinckrodt DLT	Portex DLT	Rüsch DLT	VIVASIGHT DLT	Well Lead DLT
CSA_MIDDLE_	35	114.9 (114.3–116.4)	116.9 (116.6–117.1)	105.4 (105.1–109.2; ^*∗*^87%)	107.7 (107.1–109.5)	121.3 (118.5–124.0)	117.5 (111.9–126.7)
	37	114.7 (98.6–121.7; ^*∗*^81%)	132.6 (132.1–132.6)	118.0 (114.9–121.4)	120.7 (118.4–123.6)	138.9 (137.6–139.7)	141.9 (138.3–145.2)
	39	135.9 (133.6–138.3)	152.3 (147.0–158.8)	126.9 (124.5–130.8; ^*∗*^83%)	131.7 (124.1–158.8)	146.3 (145.0–148.2)	152.0 (150.6–154.1)
	41	144.4 (142.1–147.7)	160.1 (158.0–160.4)	140.8 (139.8–143.3; ^*∗*^85%)	147.2 (145.4–149.5)	164.9 (164.3–164.9)	163.8 (162.6–164.4)

CSA_PTC_	35	126.7 (123.5–129.3)	121.1 (120.6–121.6)	118.0 (111.1–121.2)	114.3 (112.5–115.7; ^*∗*^86%)	132.2 (127.5–136.0)	130.7 (125.4–132.2)
	37	130.7 (125.6–137.0)	138.1 (136.3–140.4)	131.7 (128.6–131.9)	129.0 (126.6–130.4; ^*∗*^82%)	158.1 (153.6–163.1)	154.0 (151.1–156.5)
	39	147.1 (143.0–149.5)	148.8 (148.3–151.0)	140.8 (137.4–146.6; ^*∗*^85%)	141.5 (133.2–146.9)	165.5 (159.2–173.7)	162.9 (161.4–168.5)
	41	156.5 (154.2–157.8; ^*∗*^87%)	163.5 (163.2–163.8)	158.7 (156.1–163.0)	157.0 (154.3–158.1)	179.7 (174.0–185.8)	178.2 (171.9–182.3)

CSA_DTC_	35	135.8 (133.8–137.4)	122.6 (121.1–124.0)	120.0 (116.6–124.0)	119.5 (119.2–120.8; ^*∗*^84%)	142.7 (138.6–149.4)	129.4 (126.3–136.2)
	37	130.5 (128.2–136.6)	141.2 (139.9–142.5)	128.4 (127.0–130.8; ^*∗*^79%)	128.9 (122.6–133.1)	161.9 (158.7–166.7)	155.7 (152.0–157.2)
	39	148.8 (146.6–150.4)	152.0 (149.8–154.2)	141.2 (138.0–143.6; ^*∗*^87%)	146.6 (141.6–151.5)	162.1 (160.4–169.6)	161.2 (160.0–162.5)
	41	162.2 (158.4–165.0)	164.1 (162.7–164.9)	156.5 (154.3–161.1)	156.1 (154.7–162.1; ^*∗*^88%)	175.6 (173.7–177.6)	177.0 (173.6–178.2)

CSA_PBC_	35	74.0 (69.4–76.3)	67.1 (66.8–67.4)	65.8 (63.3–68.0; ^*∗*^85%)	77.4 (75.1–77.6)	72.9 (68.4–78.3)	72.2 (71.3–74.3)
	37	76.3 (73.3–78.9)	84.2 (81.3–86.0)	66.3 (65.5–68.6; ^*∗*^78%)	79.5 (77.5–81.1)	75.0 (72.5–77.6)	85.4 (82.5–86.3)
	39	76.7 (74.1–80.8)	89.8 (86.9–92.8)	73.9 (69.6–77.8; ^*∗*^78%)	94.3 (87.3–98.7)	78.0 (76.2–81.1)	86.6 (79.9–96.6)
	41	86.7 (83.5–93.3)	90.0 (87.2–92.3)	85.3 (83.5–90.5)	101.0 (93.0–105.3)	86.9 (85.6–87.6)	89.8 (84.5–93.4)

CSA_DBC_	35	78.3 (74.6–81.0)	72.5 (71.4–73.6)	64.3 (60.9–67.5; ^*∗*^80%)	72.9 (68.6–83.4)	80.7 (75.9–83.9)	73.0 (67.9–76.4)
	37	88.6 (86.4–90.7)	87.3 (86.4–88.3)	67.6 (66.3–68.3; ^*∗*^73%)	88.4 (77.4–93.0)	81.9 (81.1–85.5)	92.3 (87.0–93.3)
	39	81.6 (79.1–82.7; ^*∗*^86%)	94.4 (86.4–95.6)	85.7 (82.2–86.4)	91.7 (86.8–97.3)	89.8 (88.7–90.9)	91.0 (86.0–92.6)
	41	88.6 (86.4–92.3)	95.0 (93.5–97.8)	87.7 (85.5–89.9; ^*∗*^86%)	101.5 (97.4–105.8)	90.2 (86.7–92.1)	96.3 (94.2–98.0)

CSA_MIDDLE_ = cross-sectional area in the middle (B) between the beginning of the DLT shaft (A) and the proximal edge of the tracheal cuff (C); CSA_PTC_ = cross-sectional area at the proximal edge of the tracheal cuff (C); CSA_DTC_ = cross-sectional area at the distal edge of the tracheal cuff (D); CSA_PBC_ = cross-sectional area at the proximal edge of the bronchial cuff (E); CSA_DBC_ = cross-sectional area at the distal edge of the bronchial cuff (F) (see also Figure 1). DLT sizes are in French (= 1/3 mm). Data are displayed as median (minimum-maximum) given as millimeter [2]. The largest CSA of a respective DLT size is shaded in gray (100%), and the smallest diameter marked is with an asterisk (^*∗*^) (proportion of largest OD).

**Table 4:** Measured length of investigated left-sided double-lumen endobronchial tubes (DLTs).

Parameter	Size (Fr)	Sheridan DLT	Mallinckrodt DLT	Portex DLT	Rüsch DLT	VIVASIGHT DLT	Well Lead DLT
A–C	35	260.0 (254.0–261.0)	253.0 (252.0–254.0)	318.5 (315.0–322.0)	210.0 (207.0–214.0; ^*∗*^66%)	241.0 (235.0–247.0)	250.0 (248.0–253.0)
	37	258.0 (256.0–261.0)	250.5 (249.0–252.0)	317.5 (316.0–319.0)	215.0 (214.0–215.0; ^*∗*^68%)	241.0 (241.0–243.0)	252.5 (248.0–256.0)
	39	263.0 (262.0–263.0)	248.0 (247.0–248.5)	307.5 (304.0–308.0)	234.5 (233.0–236.0; ^*∗*^76%)	241.0 (238.0–243.0)	251.0 (246.0–256.0)
	41	264.5 (264.0–265.0)	249.5 (247.0–251.0)	304.5 (301.0–308.0)	235.0 (234.0–236.0; ^*∗*^77%)	242.0 (239.0–246.0)	243.0 (239.0–247.0)

C-D	35	37.3 (36.0–40.0)	40.0 (40.0–40.0)	32.0 (32.0–32.8; ^*∗*^73%)	36.3 (36.0–37.0)	44.0 (43.7–44.3)	44.0 (43.0–45.0)
	37	36.8 (35.5–38.0; ^*∗*^86%)	43.0 (42.0–43.5)	38.1 (37.0–39.0)	37.0 (35.0–39.5)	41.9 (41.7–43.0)	43.0 (42.0–44.0)
	39	38.3 (38.0–39.0)	43.0 (42.0–44.0)	39.0 (37.0–42.0)	37.5 (36.5–39.0; ^*∗*^83%)	45.2 (45.0–45.5)	42.0 (41.0–42.5)
	41	38.0 (37.0–40.0; ^*∗*^86%)	39.0 (38.0–39.1)	42.9 (41.0–44.0)	38.1 (38.0–39.0)	44.2 (43.3–46.0)	42.3 (42.0–43.0)

D-E	35	38.0 (36.0–40.0)	35.0 (35.0–35.0)	40.0 (39.0–41.0)	34.5 (31.0–38.0:^*∗*^78%)	36.5 (35.6–37.0)	44.5 (39.0–48.0)
	37	43.5 (42.0–45.0)	34.3 (34.0–35.0; ^*∗*^79%)	39.5 (37.0–42.0)	40.5 (39.0–41.0)	40.8 (40.0–41.0)	42.5 (38.0–46.0)
	39	48.0 (45.0–51.0)	40.0 (38.5–42.0)	38.5 (35.0–41.0; ^*∗*^80%)	42.0 (40.0–43.0)	39.0 (37.0–40.3)	44.5 (40.0–48.0)
	41	42.0 (38.0–45.0)	47.0 (44.0–49.0)	39.0 (36.0–42.0; ^*∗*^76%)	52.0 (51.0–53.0)	40.3 (39.0–41.8)	51.5 (51.0–52.0)

E-F	35	23.0 (23.0–23.5)	16.8 (16.5–17.0)	24.5 (23.0–25.3)	17.4 (16.0–18.3)	14.9 (14.3–15.0; ^*∗*^61%)	15.7 (15.4–16.1)
	37	22.5 (22.0–23.0)	19.5 (19.0–20.0)	25.4 (24.8–27.0)	16.6 (16.0–18.0)	17.1 (17.0–17.6)	15.9 (15.1–16.5; ^*∗*^63%)
	39	18.0 (18.0–18.3)	18.5 (17.5–19.0)	30.0 (30.0–30.3)	19.0 (18.0–19.0)	17.3 (16.0–17.6)	15.8 (15.0–16.0; ^*∗*^53%)
	41	21.5 (21.0–22.0)	17.1 (17.0–18.0)	30.8 (30.0–31.0)	16.5 (16.3–17.0)	17.1 (17.0–17.5)	15.6 (15.0–16.1; ^*∗*^51%)

F-G	35	8.1 (7.0–9.5)	11.1 (10.9–11.3)	4.6 (4.0–5.7; ^*∗*^28%)	6.0 (5.0–6.5)	16.4 (15.5–17.0)	11.2 (10.3–11.5)
	37	8.0 (7.3–8.1)	12.0 (11.5–12.1)	4.8 (4.5–4.8; ^*∗*^32%)	7.1 (6.0–7.6)	14.9 (12.5–18.0)	12.0 (11.9–12.7)
	39	10.0 (9.5–10.0)	11.7 (11.1–11.9)	6.6 (6.3–6.8)	6.0 (5.0–6.1; ^*∗*^46%)	13.1 (12.9–14.0)	10.6 (10.5–11.0)
	41	8.6 (8.0–9.0)	14.2 (14.0–14.4)	7.0 (6.8–7.5; ^*∗*^48%)	7.5 (7.0–8.0)	14.6 (14.0–15.0)	10.5 (10.0–11.7)

A–G	35	366.4 (356.0–374.0)	355.9 (354.4–357.3)	419.6 (413.0–426.7)	304.1 (295.0–313.8; ^*∗*^72%)	352.7 (344.1–360.3)	365.4 (355.6–373.6)
	37	368.8 (362.8–375.1)	359.3 (355.5–362.6)	425.3 (419.3–431.8)	316.2 (310.0–321.1; ^*∗*^74%)	355.6 (352.1–362.6)	365.9 (355.0–375.2)
	39	377.3 (372.5–381.3)	361.2 (356.1–365.4)	421.6 (412.3–428.0)	339.0 (332.5–343.1; ^*∗*^80%)	355.5 (348.9–360.4)	363.8 (352.5–373.5)
	41	374.6 (368.0–381.0)	366.8 (360.0–371.5)	424.1 (414.8–432.5)	349.1 (346.3–353.0; ^*∗*^82%)	358.2 (352.3–366.3)	362.8 (357.0–369.8)

A–C = distance from bifurcation (beginning of DLT shaft, A) to the proximal edge of the tracheal cuff (C); C-D = length of the tracheal cuff; D-E = distance between distal edge of the tracheal cuff (D) to the proximal edge of the bronchial Cuffs (E); E-F = length of the bronchial cuff; F-G = distance between the distal edge of the bronchial Cuffs (F) to the tip of the tube (G); A–G = tube length from bifurcation (A) to the tip of the tube (G) (see also Figure 1). DLT sizes are in French (= 1/3 mm). Data are displayed as median (minimum–maximum) given as millimeter (mm). The greatest length of a respective DLT size is shaded in gray (100%), and the smallest diameter is marked with an asterisk (^*∗*^) (proportion of largest OD).

**Table 5:** Tracheal and bronchial outer cuff diameters of the investigated left-sided double-lumen endobronchial tubes (DLTs) measured at a cuff pressure of 20 cm H_2_O.

Parameter	Size (Fr)	Sheridan DLT	Mallinckrodt DLT	Portex DLT	Rüsch DLT	VIVASIGHT DLT	Well Lead DLT
Tracheal cuff	35	23.4 (23.3–23.7; ^*∗*^78%)	24.3 (24.3–24.3)	28.5 (28.3–29.2)	27.6 (26.9–28.4)	28.3 (27.9–28.6)	29.7 (29.0–30.2)
	37	25.9 (25.7–26.3)	25.0 (24.1–26.4; ^*∗*^78%)	32.1 (31.7–32.7)	27.0 (26.7–27.4)	28.6 (27.9–29.3)	28.9 (27.7–29.0)
	39	24.9 (24.4–25.0; ^*∗*^77%)	26.3 (26.2–26.5)	32.2 (31.9–33.0)	28.6 (27.3–29.6)	28.8 (28.7–29.1)	29.6 (28.7–30.1)
	41	24.7 (24.5–24.9; ^*∗*^72%)	25.6 (25.0–26.0)	34.2 (33.3–34.4)	27.1 (27.0–28.0)	29.3 (29.1–29.6)	30.5 (29.7–31.0)

Bronchial cuff	35	19.3 (19.0–19.8)	20.6 (20.4–20.8)	19.6 (19.3–20.2)	13.5 (12.5–14.4; ^*∗*^66%)	18.2 (17.6–18.7)	18.6 (18.4–19.2)
	37	19.1 (18.9–20.1)	20.3 (19.5–21.0)	20.2 (19.5–20.7)	13.8 (13.4–14.4; ^*∗*^68%)	17.2 (16.4–18.2)	19.2 (17.9–19.7)
	39	19.9 (19.8–20.1)	19.4 (19.0–20.0)	24.6 (24.0–26.0)	16.7 (16.4–17.0; ^*∗*^68%)	24.2 (19.0–28.8)	18.8 (18.4–19.1)
	41	18.2 (17.8–18.6)	20.3 (20.0–20.9)	26.0 (25.0–27.0)	14.0 (13.6–14.5; ^*∗*^54%)	18.7 (18.2–19.2)	19.1 (18.8–19.4)

DLT sizes are in French (= 1/3 mm). Data are displayed as median (minimum-maximum) given as millimeter (mm). The largest cuff diameter of a respective DLT size is shaded in gray (100%), and the smallest diameter is marked with an asterisk (^*∗*^) (proportion of largest OD).

**Table 6:** Measured angles of the investigated left-sided double-lumen endobronchial tubes (DLTs) between a virtual longitudinal line through the middle of the tracheal tube shaft and bronchial tube (see also Figure 1).

Parameter	Size (Fr)	Sheridan DLT	Mallinckrodt DLT	Portex DLT	Rüsch DLT	VIVASIGHT DLT	Well Lead DLT
Angle	35	39 (35–40)	12 (12–12)	40 (37–46)	34 (32–34)	26 (25–27)	37 (36–40)
	37	28 (26–32)	20 (18–22)	39 (34–42)	28 (27–30)	24 (23–24)	35 (34–36)
	39	26 (22–29)	18 (18–20)	39 (36–43)	27 (24–28)	43 (40–47)	35 (34–36)
	41	31 (29–34)	27 (25–28)	37 (36–38)	30 (28–32)	46 (42–51)	38 (36–40)

DLT sizes are in French (= 1/3 mm). Data is displayed as median (minimum-maximum) and dimension is degrees (°).

## Data Availability

All data on which the conclusions of the manuscript rely are presented in the main paper.

## Abbreviations

CSA: Cross-sectional area
CT: Computed tomography
DLT: Double-lumen endobronchial tube
Fr: French (1/3 mm)
ID: Internal diameter
OD: Outer diameter
Ch: Charriere
F: French (1/3 mm)
ISO: International Organization for Standardization.
